# A Cohort Study of the Milk Microbiota of Healthy and Inflamed Bovine Mammary Glands From Dryoff Through 150 Days in Milk

**DOI:** 10.3389/fvets.2018.00247

**Published:** 2018-10-09

**Authors:** Stephanie A. Metzger, Laura L. Hernandez, Joseph H. Skarlupka, Teresa M. Walker, Garret Suen, Pamela L. Ruegg

**Affiliations:** ^1^Department of Dairy Science, University of Wisconsin-Madison, Madison, WI, United States; ^2^Department of Bacteriology, University of Wisconsin-Madison, Madison, WI, United States

**Keywords:** milk microbiome, milk microbiota, mastitis, somatic cell count, 16S sequencing

## Abstract

The objective of this longitudinal cohort study was to describe the milk microbiota of dairy cow mammary glands based on inflammation status before and after the dry period. Individual mammary quarters were assigned to cohorts based on culture results and somatic cell count (**SCC**) at dryoff and twice in the first 2 weeks post-calving. Mammary glands that were microbiologically negative and had low SCC (< 100,000 cells/mL) at all 3 sampling periods were classified as Healthy (*n* = 80). Microbiologically negative mammary glands that had SCC ≥150,000 cells/mL at dryoff and the first post-calving sample were classified as Chronic Culture-Negative Inflammation (CHRON; *n* = 17). Quarters that did not have both culture-negative milk and SCC ≥ 150,000 cells/mL at dryoff but were culture-negative with SCC ≥ 150,000 at both post-calving sampling periods were classified as Culture-Negative New Inflammation (NEWINF; *n* = 6). Mammary glands with bacterial growth and SCC ≥ 150,000 cells/mL at all 3 periods were classified as Positive (POS; *n* = 3). Milk samples were collected from all enrolled quarters until 150 days in milk and subjected to microbiota analysis. Milk samples underwent total DNA extraction, a 40-cycle PCR to amplify the V4 region of the bacterial 16S rRNA gene, and next-generation sequencing. Healthy quarters had the lowest rate of PCR and sequencing success (53, 67, 83, and 67% for Healthy, CHRON, NEWINF, and POS, respectively). Chao richness was greatest in milk collected from Healthy quarters and Shannon diversity was greater in milk from Healthy and CHRON quarters than in milk collected from glands in the NEWINF or POS cohorts. Regardless of cohort, season was associated with both richness and diversity, but stage of lactation was not. The most prevalent OTUs included typical gut- and skin-associated bacteria such as those in the phylum Bacteroidetes and the genera *Enhydrobacter* and *Corynebacterium*. The increased sequencing success in quarters with worse health outcomes, combined with the lack of bacterial growth in most samples and the high PCR cycle number required for amplification of bacterial DNA, suggests that the milk microbiota of culture-negative, healthy mammary glands is less abundant than that of culture-negative glands with a history of inflammation.

## Introduction

Mastitis, or inflammation of the mammary gland, is a common disease of dairy cattle that causes decreased milk production (1, 2). Mastitis is characterized based on the magnitude of the inflammatory response and is classified as clinical mastitis (**CM**), when milk or the udder has visible abnormalities; or subclinical mastitis (**SM**), when milk appears visually normal but the somatic cell count (**SCC**) exceeds normal levels. Producers often collect milk samples from affected mammary glands for culture-based microbiological analysis to determine the etiology (3), but many samples result in no growth in aerobic culture (4–6). As such, culture-negative, visually normal milk with a low SCC (< 100,000 cells/mL) is often considered healthy and was previously considered sterile due to the lack of growth in culture (7).

In recent years, as researchers have identified the importance of the microbiota in other dairy cow organ systems (8, 9), culture-independent sequencing techniques have been applied to culture-negative milk samples with the objective of determining if milk has a microbiota similar to that of other systems and if the milk contains pathogens undetectable by traditional means. These reports indicate that healthy milk contains DNA from bacteria not previously associated with milk, such as members of the family Lachnospiraceae and genera *Faecalibacterium* (10, 11) and *Enhydrobacter* (12). Early studies also suggest that milk samples obtained from presumably healthy mammary glands have greater bacterial richness and diversity, as compared to the microbiota of milk collected from glands experiencing CM (10, 13, 14). However, the concept of a healthy milk microbiota has been questioned due to the physiology of the mammary gland and the low concentration of viable bacteria or bacterial DNA in milk collected from apparently healthy glands (15).

In intensive dairy systems of the Northern Hemisphere, the risk of mastitis is associated with cow characteristics such as parity (older cows are at greater risk), stage of lactation (earlier lactation has greater risk), and season (cows are at greater risk in summer) (16–18) and the associated microbiota composition may also be correlated to these factors. However, prior reports of the milk microbiota have often not included descriptions of the cow population or environment (10, 13, 14). For example, bedding is a major source of bacterial exposure for the mammary gland, and a cross-sectional study of the milk microbiota in relation to bedding type found that, although diversity did not differ by bedding type, there were differences with respect to overall community composition (12). Age is also another likely factor, as older cows are more susceptible to mastitis (16, 18), and milk samples collected from glands with CM often have lower bacterial richness and diversity than milk samples collected from apparently healthy glands; however, associations between parity and microbiota status are yet unknown.

A significant challenge in understanding the milk microbiota, as it relates to mastitis, is that previous work has not tracked this microbiota longitudinally, even though mastitis is a temporal condition. In other mammals such as humans, the milk microbiota has been reported to change across the first 6 months of lactation (19), but the longest study reported to date of the milk microbiota of cows is 2 weeks (14). In that study, the richness and diversity of apparently healthy milk did not change across those 2 weeks, but the SCC and culture results of the milk were not reported (14), rendering the actual health status of the mammary quarters indeterminate. To address this apparent gap in knowledge, we undertook a prospective longitudinal cohort study to describe the milk microbiota from bovine mammary quarters from dryoff through the first 150 days of the next lactation. Somatic cell count and microbiological status were assessed for each of these quarters prior to dryoff and milk samples were subjected to 16S rDNA microbiota sequencing. Our study represents the first extensive longitudinal study of the milk microbiota.

## Materials and methods

### Quarter selection and enrollment

This study was approved and performed based on University of Wisconsin-Madison IACUC protocol A01548-08-13. All mammary gland quarters of all cows completing a lactation at the University of Wisconsin-Madison dairy research herd were screened for potential enrollment at the final milking prior to dryoff (**DR**), at 4–7 days in milk (**DIM**) of the next lactation (**C1**), and again at 11-14 DIM (**C2**). Milk samples were collected by researchers for microbiological analysis and SCC determination (CombiFOSS 6000, Foss Food Technology Corp., Hillerød, Denmark). Milk samples from quarters enrolled into predefined cohorts were collected weekly for SCC and every 4 weeks for microbiota analysis until the cows reached 150 DIM. Individual quarters within a cow were assigned to cohorts based on SCC and culture status at the DR, C1, and C2 samples (Table S1). Quarters that had a low SCC (< 100,000 cells/mL) and no growth at all 3 sampling periods were assigned to the Healthy cohort. Quarters with SCC ≥ 150,000 cells/mL but no bacterial growth from milk samples collected at DR and C1 were classified as chronically inflamed (CHRON). Quarters with a variable DR sample and SCC ≥ 150,000 cells/mL without bacterial growth in milk samples collected at both C1 and C2 were classified as having new culture-negative inflammation (NEWINF). Quarters with bacterial growth and SCC ≥ 150,000 cells/mL at DR, C1, and C2 were classified as positive (POS). After quarters were enrolled in a cohort, weekly milk samples were collected for determination of SCC and aseptic milk samples were collected for microbiological analysis monthly until quarters were 150 DIM (**M2**, **M3**, **M4**, and **M5**). The herd used computerized health records for all animals (Dairy Comp 305, Valley Agricultural Systems, Tulare, CA) and lactating cows were housed in sand-bedded freestall barns. Some cows (*n* = 12) were transferred to a different sawdust-bedded tiestall facility within the same herd from calving through early lactation. Sample collection began in February 2014 and ended in July 2015.

### Milk sample collection and culturing

Milk samples were aseptically collected by researchers in the milking parlor immediately prior to the morning milking. Research personnel wiped udders with a clean, dry cloth to remove bedding and visible contaminants. Milking personnel then performed standard pre-milking sanitation: 2–3 streams of foremilk per teat were discarded, then a 0.5% iodine predip solution (Theratec Plus, GEA, Columbia, MD) was applied, 30–60 s contact time was allowed, and teats were dried with a dry cloth towel. The researcher then put on clean nitrile gloves, discarded 2–3 streams of milk, scrubbed the teat with 70% isopropanol, allowed the isopropanol to dry, discarded another 2–3 streams of milk, and collected approximately 40 mL milk into a sterile sample vial. Next, 40 mL milk was collected into a nonsterile plastic vial containing a bronopol tablet for later SCC analysis. New gloves were used for aseptic collection of milk from each mammary gland. In accordance with normal herd health protocols, after the dryoff milking, every quarter was infused with an intramammary antimicrobial containing 1,000,000 IU penicillin and 1.0 g dihydrostreptomycin (Quartermaster, West Agro, Inc., Hamilton, NY) and administered a teat sealant containing 4 g bismuth subnitrate (Orbeseal, Zoetis, Parsippany, NJ).

All milk samples were placed on ice and transported to the laboratory to be cultured within 12 h of collection following National Mastitis Council procedures (20). One hundred microliter of milk were inoculated on half of a plate containing trypticase soy agar with 5% sheep blood (BD, Franklin Lakes, NJ) and half of a MacConkey agar plate (BD, Franklin Lakes, NJ). Inoculated agars were incubated aerobically at 37°C for 48 h. Samples with 0 or 1 colony at 48 h were considered negative. Samples with >2 colonies of a single type were considered positive and subjected to further biochemical testing. Samples with more than 2 colony types, regardless of the number of colonies, were considered contaminated (20). For positive samples, a single colony was selected for biochemical testing. Colonies were Gram-stained and examined with brightfield microscopy for cellular morphology. Gram-positive colonies were tested for catalase production; catalase-positive colonies were then tested for coagulase production and mannitol salt agar reaction while catalase-negative colonies were tested for triple sugar iron agar reaction, citrate utilization, motility, indole production, and ornithine production.

### DNA extraction, PCR, and sequencing

DNA was extracted from milk samples using buffers from a QIAamp DNA Stool Mini Kit (Qiagen, Frederick, MD) as previously described (12). Four milliliters whole milk was centrifuged at 13,000 × *g* for 20 min at 4°C. The milk fat layer and supernatant were discarded. Buffer ASL was added and samples were frozen and thawed 5 × with liquid N_2_ and a 37°C water bath. Samples were then incubated with lysozyme for 30 min in a 37°C water bath. Next, Proteinase K and Buffer ASL were added for 10 min and then absolute ethanol was added. Samples were transferred to spin columns, washed with Buffer PE, and eluted with Buffer AE. Eluted DNA was lyophilized, suspended in 20 μL nuclease-free water, and quantified using a Qubit (ThermoFisher, Waltham, MA).

DNA was diluted to 0.625 ng/μL and 8 μL DNA was added to each PCR reaction along with 6.6 μL Phusion Master Mix (New England BioLabs, Ipswich, MA), 1.0 μL of 10 μM forward universal bacterial barcoded primer (5′-GTGCCAGCMGCCGCGGTAA-3′), and 1.0 μL of 10 μM reverse universal bacterial barcoded primer (5′-GGACTACHVGGGTWTCTAAT-3′) for the 16S V4 region. Nuclease-free water was added to bring the reaction volume to 20 μL. The initial PCR denaturing step was 30 s at 98°C, and was followed by 8 s of denaturing at 98°C, 20 s of annealing at 58°C, and a 20-s extension step at 72°C. Forty PCR cycles were performed, and the PCR was completed with 5 min of extension at 72°C. Negative controls of nuclease-free water were subjected to PCR and sequencing along with experimental samples.

All PCR products were visualized on an agarose gel. Visible bands were excised from the gel and extracted with a Zymoclean gel DNA recovery kit (Zymo Research, Irvine, CA). After gel extraction, DNA was quantified with high-sensitivity Qubit reagents and pooled at equimolar concentrations prior to sequencing with 10% PhiX control DNA on an Illumina MiSeq (San Diego, CA) (9). Raw sequences were obtained in fastq format and cleaned using mothur v 1.38.1 (21) as described previously (12). Sequences were aligned to the SILVA 16S rRNA gene reference database (Release 128) (22). All sequences have been deposited in the National Center for Biotechnology Information's Sequence Read Archive under BioProject ID PRJNA478482.

### Statistical analysis

Individual mammary quarters were the experimental units analyzed using SAS 9.4 (Cary, NC). Sample collection dates were used to classify data by season (fall: Sept.-Nov.; winter: Dec.-Feb.; spring: Mar.-May; summer: Jun.-Aug.). Parity was based on lactation at the subsequent calving (not DR). Sequencing success was defined as having a visible PCR amplicon band that produced sequence reads on an Illumina MiSeq. To test the hypothesis that sequencing success did not differ among cohorts, a logistic model was constructed in Proc LOGISTIC with sequencing success as the outcome variable. The explanatory variables parity (2, 3, 4–7), facility at time of sampling (sand-bedded, sawdust-bedded), sampling period (DR, C1, C2, M2, M3, M4, M5), and season (fall, winter, spring, summer) were tested for significance and variables with greatest *P*-values were eliminated in a backwards stepwise manner until only variables with *P*-values ≤ 0.05 remained. To test the hypotheses that Chao richness or Shannon diversity did not differ among cohorts, regression models were constructed in Proc MIXED with the same explanatory variables and backwards stepwise elimination as in the logistic model. Canonical discriminant analysis was performed in Proc CANDISC using the relative abundances of operational taxonomic units (**OTUs**).

## Results

### Herd characteristics and enrolled quarters

The herd consisted of approximately 665 lactating cows with a rolling herd average production of 12,100 kg milk and a bulk tank SCC of approximately 180,000 cells/mL. A total of 1,078 quarters from 270 cows were screened for enrollment at dryoff and twice in the first 2 weeks of the next lactation. Because the first sample period was at the final milking of a lactation, every quarter was parity 2 or greater at the subsequent calving. No cows were sampled during the first 150 DIM of the first lactation. Twelve cows (16 enrolled quarters) were transferred to the sawdust-bedded facility within the university herd for part of the subsequent lactation. The Healthy cohort contained 80 quarters from 55 cows, the CHRON cohort had 17 quarters from 14 cows, the NEWINF cohort had 6 quarters from 6 cows, and the POS cohort had 3 quarters from 3 cows (Table 1). Quarters enrolled to the Healthy cohort were from cows that were younger than cows enrolled to the CHRON, NEWINF, or POS cohorts (Table 1). Milk collected from cows in the Healthy cohort had the lowest SCC throughout lactation, while milk from cows enrolled in the CHRON cohort had low SCC after the C1 sample. Milk from cows enrolled in both the NEWINF and POS cohorts had increased SCC throughout the enrollment and follow-up periods (*P* < 0.0001) (Figure S1) with more CM (*P* < 0.0001) (Figure S2). Three cows, each with one quarter in the Healthy cohort, one cow with one quarter in the CHRON cohort, and a cow with one quarter from the NEWINF cohort died during the study period.

**Table 1:** Descriptive statistics of enrolled bovine mammary glands.

**Cohort**	***n***	**Parity**	**Previous clinical mastitis^a^ (*n*)**	**DR^b^ SCC^c^**	**C1 SCC**	**C2 SCC**	**Incident clinical mastitis cases (*n*)^d^**	**Incident positive monthly culture cases (*n*)^e^**
Healthy	80	3	5	4.45	4.46	4.17	2	8
CHRON	17	4	5	5.72	5.45	4.68	3	6
NEWINF	6	4	2	5.42	5.80	5.69	3	6
POS	3	5	0	5.69	5.98	5.96	1	3
Total	106	4	12	5.66	5.59	5.06	9	23

a Quarters that were treated for clinical mastitis with an intramammary antimicrobial during the previous lactation according to herd records.

b *DR, dryoff, C1, first week of lactation, C2, second week of lactation*.

c *All values are the log_10_SCC*.

d *Number of quarters that developed clinical mastitis during the follow-up period according to herd records*.

e *Number of quarters that had an aseptic milk sample with positive bacterial growth at one or more monthly milk samples*.

*Healthy, Quarters that had culture-negative milk with SCC < 100,000 cells/mL at DR, C1, and C2; CHRON, Chronically inflamed quarters that had culture-negative milk samples with SCC ≥ 150,000 cells/mL at DR and C1; NEWINF, Quarters that had new culture-negative inflammation with variable DR milk sample results and culture-negative milk samples with SCC ≥ 150,000 cells/mL at C1 and C2; POS, Quarters that had bacterial growth in culture and SCC ≥ 1,50,000 cells/mL at DR, C1, and C2*.

### DNA extraction and PCR success

**Figure 1:**
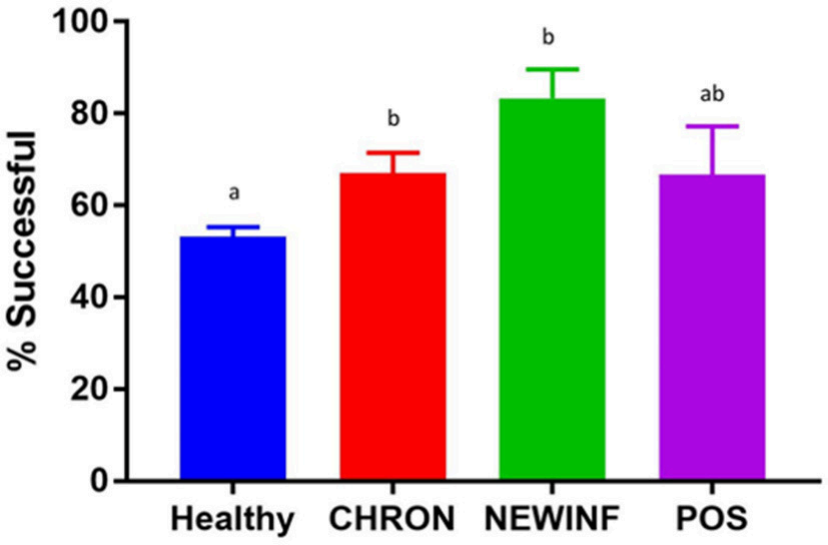
Proportion of successful PCR and sequencing by cohort. Healthy, Quarters (*n* = 80) that had culture-negative milk with SCC < 100,000 cells/mL at DR, C1, and C2. CHRON, Quarters (*n* = 17) that had culture-negative milk samples with SCC ≥ 150,000 cells/mL at DR and C1; NEWINF, Quarters (*n* = 6) that had new culture-negative inflammation with variable DR milk sample results and culture-negative milk samples with SCC ≥ 150,000 cells/mL at C1 and C2; POS, Quarters (*n* = 3) that had bacterial growth in culture and SCC ≥ 150,000 cells/mL at DR, C1, and C2; Sampling periods, DR, dryoff, C1; first week of lactation; C2, second week of lactation. a,b Columns with different superscripts differ (*P* < 0.05).

In total, 723 milk samples were collected for microbiota analysis. Seventeen samples had insufficient sample volume for DNA extraction and 14 samples collected on a single day were erroneously discarded prior to extraction. Of the 692 samples subjected to PCR and sequencing, 397 (57.4%) were successful (Table 2). Sequencing success ranged from 53.2% in Healthy samples to 83.3% in NEWINF samples (*P* < 0.001) (Figure 1). Milk samples from NEWINF and CHRON quarters were 4.56 times more likely (95% CI: 1.85–11.2) and 1.79 times more likely (95% CI: 1.16–2.77) to have sequencing success, as compared to milk samples collected from Healthy quarters (*P* < 0.001) (Table S2). The odds of successful sequencing were greater for milk samples collected at M5 as compared to milk samples collected at any other time point (*P* = 0.044) (Table S2).

**Table 2:** Sequencing success by cohort and sampling period.

	**DR**	**C1**	**C2**	**M2**	**M3**	**M4**	**M5**
Healthy	39/79	44/73	46/77	30/71	46/76	29/75	44/72
CHRON	13/17	9/15	9/17	10/15	13/17	9/15	12/16
NEWINF	3/4	6/6	3/5	5/5	5/6	4/6	4/4
POS	2/3	2/3	1/3	3/3	1/3	3/3	2/3

*Healthy, Quarters (n = 80) that had culture-negative milk with SCC < 100,000 cells/mL at DR, C1, and C2; CHRON, Quarters (n = 17) that had culture-negative milk samples with SCC ≥ 150,000 cells/mL at DR and C1; NEWINF, Quarters (n = 6) that had new culture-negative inflammation with variable DR milk sample results and culture-negative milk samples with SCC ≥ 150,000 cells/mL at C1 and C2; POS, Quarters (n = 3) that had bacterial growth in culture and SCC ≥ 150,000 cells/mL at DR, C1, and C2; Sampling periods, DR, dryoff, C1, first week of lactation, C2, second week of lactation, M2, second month of lactation, M3, third month of lactation, M4, fourth month of lactation, M5, fifth month of lactation*.

### Microbiota

#### Sequences and diversity

**Figure 2:**
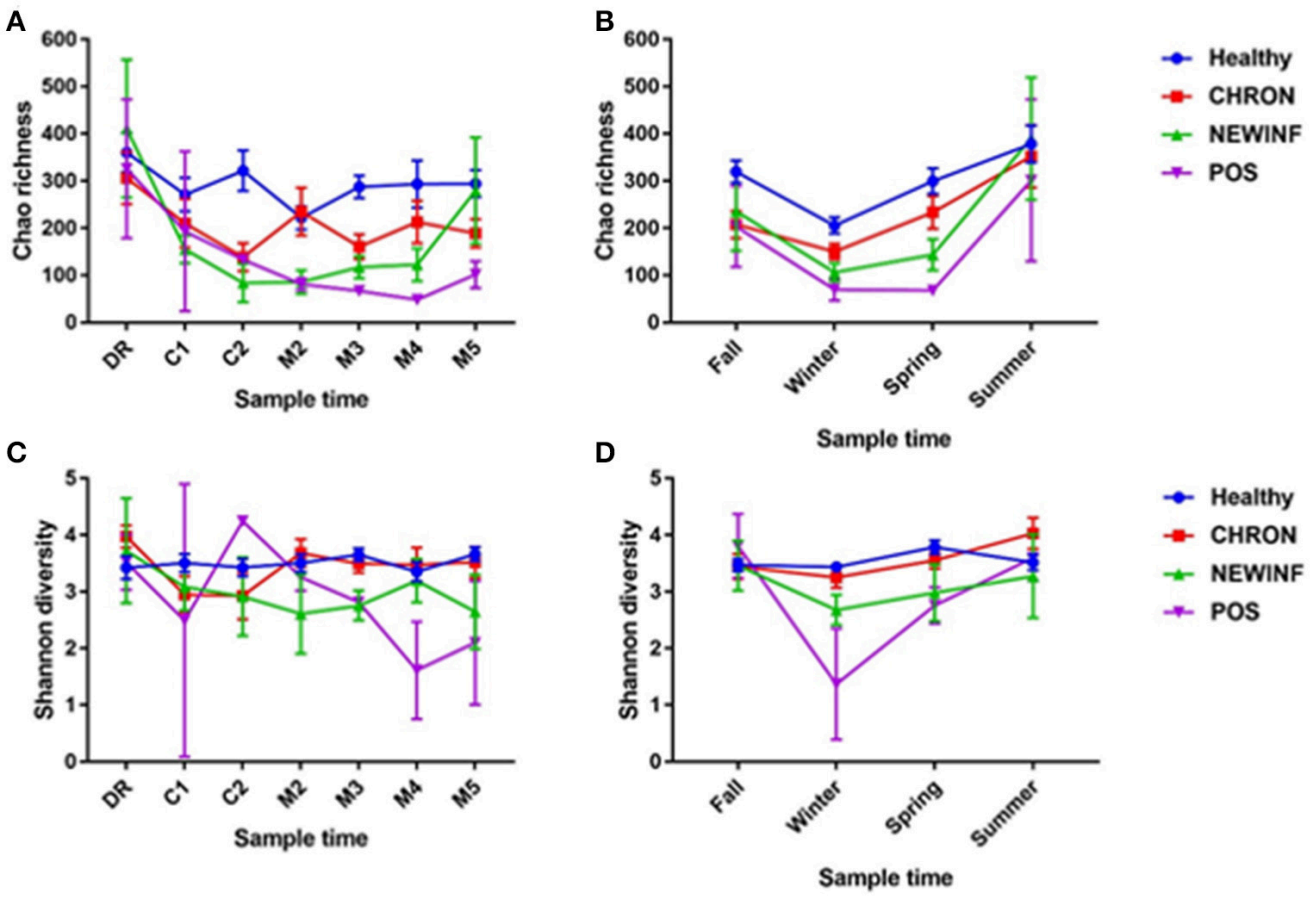
Richness across lactation **(A)** and by season **(B)** and diversity across lactation **(C)** and by season **(D)** for each cohort. Healthy, Quarters that had culture-negative milk with SCC < 100,000 cells/mL at DR, C1, and C2. CHRON, Chronically inflamed quarters that had culture-negative milk samples with SCC ≥ 150,000 cells/mL at DR and C1. NEWINF, Quarters that had new culture-negative inflammation with variable DR milk sample results and culture-negative milk samples with SCC ≥ 150,000 cells/mL at C1 and C2. POS, Quarters that had bacterial growth in culture and SCC ≥ 150,000 cells/mL at DR, C1, and C2. DR, dryoff; C1, first week of lactation; C2, second week of lactation; M2, second month of lactation; M3, third month of lactation; M4, fourth month of lactation; M5, fifth month of lactation.

A total of 26,442,947 raw reads were generated for an average of 66,606 reads per sample. 10,256,631 reads (25,835 reads/sample) were retained after cleanup. Due to the large number of PCR cycles required to amplify DNA, reads matching those found in our negative controls (5 total OTUs) were removed from all sample results (Figure S3). Data were then normalized to 3,000 sequences per sample, which represents the lowest sequence amount for all samples, and a sequence clustering analysis of the normalized samples produced 11,304 OTUs. Chao richness was determined for these samples with cohort, season, and parity group retained in the final regression model due to significance, while sampling period was forced into the model. Chao richness was greater in Healthy quarters than other quarters (*P* = 0.016) and did not differ based on sampling period (*P* = 0.38) (Figure 2A) but increased from winter to summer (*P* = < 0.0001) (Figure 2B). Shannon diversity was greater in milk samples collected from Healthy and CHRON quarters as compared to milk samples collected from NEWINF and POS quarters (*P* = 0.0019). Stage of lactation was not associated with Shannon diversity (*P* = 0.77) (Figure 2C) and Shannon diversity tended to be greater in spring than in winter (*P* = 0.089) (Figure 2D).

#### Prevalent OTUs

**Figure 3:**
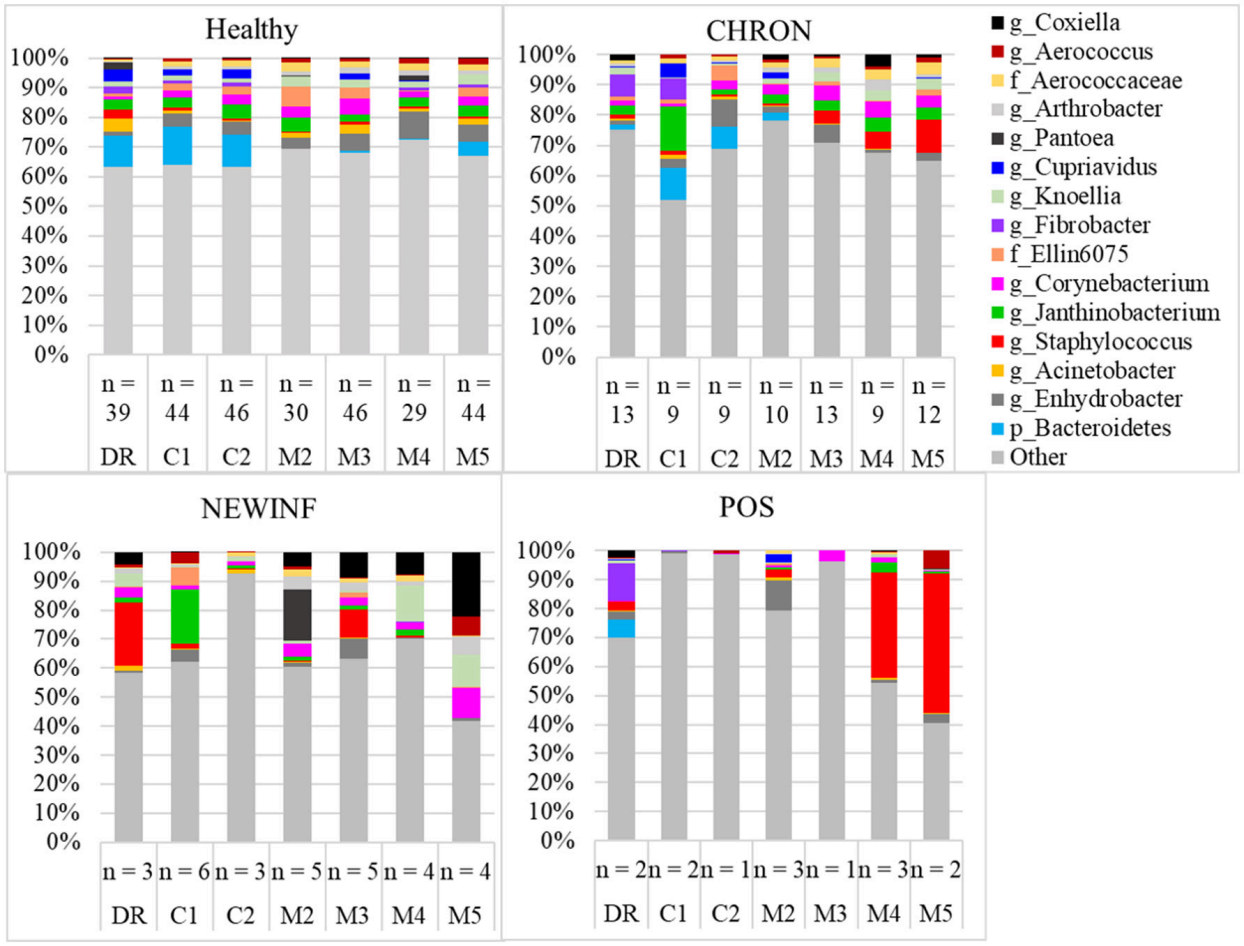
Relative abundance of OTUs in milk samples from each cohort. Healthy, Quarters that had culture-negative milk with SCC < 100,000 cells/mL at DR, C1, and C2; CHRON, Chronically inflamed quarters that had culture-negative milk samples with SCC ≥ 150,000 cells/mL at DR and C1; NEWINF, Quarters that had new culture-negative inflammation with variable DR milk sample results and culture-negative milk samples with SCC ≥ 150,000 cells/mL at C1 and C2; POS, Quarters that had bacterial growth in culture and SCC ≥ 150,000 cells/mL at DR, C1, and C2; DR, dryoff, C1, first week of lactation, C2, second week of lactation, M2, second month of lactation, M3, third month of lactation, M4, fourth month of lactation, M5, fifth month of lactation.

**Figure 4:**
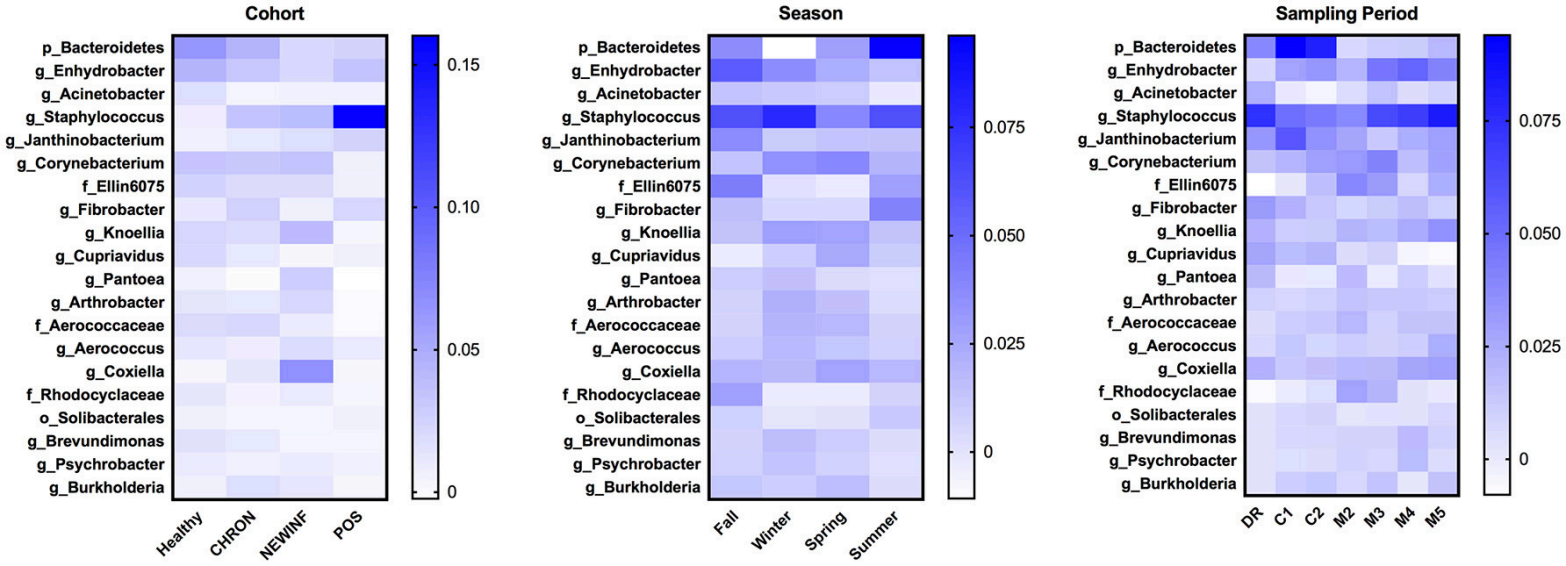
Heat maps of the relative abundances of the 20 most prevalent OTUs in milk samples by cohort, season, or sampling period. Values included in the heat maps are LSM estimates generated from the models containing cohort, season, and sampling period as predictor variables for the prediction of the proportion of sequences represented by each OTU. The predicted values are included in the heat maps, even when the predicted proportion of sequences is < 0. Healthy, Quarters that had culture-negative milk with SCC < 100,000 cells/mL at DR, C1, and C2; CHRON, Chronically inflamed quarters that had culture-negative milk samples with SCC ≥ 150,000 cells/mL at DR and C1; NEWINF, Quarters that had new culture-negative inflammation with variable DR milk sample results and culture-negative milk samples with SCC ≥ 150,000 cells/mL at C1 and C2; POS, Quarters that had bacterial growth in culture and SCC ≥ 150,000 cells/mL at DR, C1, and C2; DR, dryoff, C1, first week of lactation, C2, second week of lactation.

**Figure 5:**
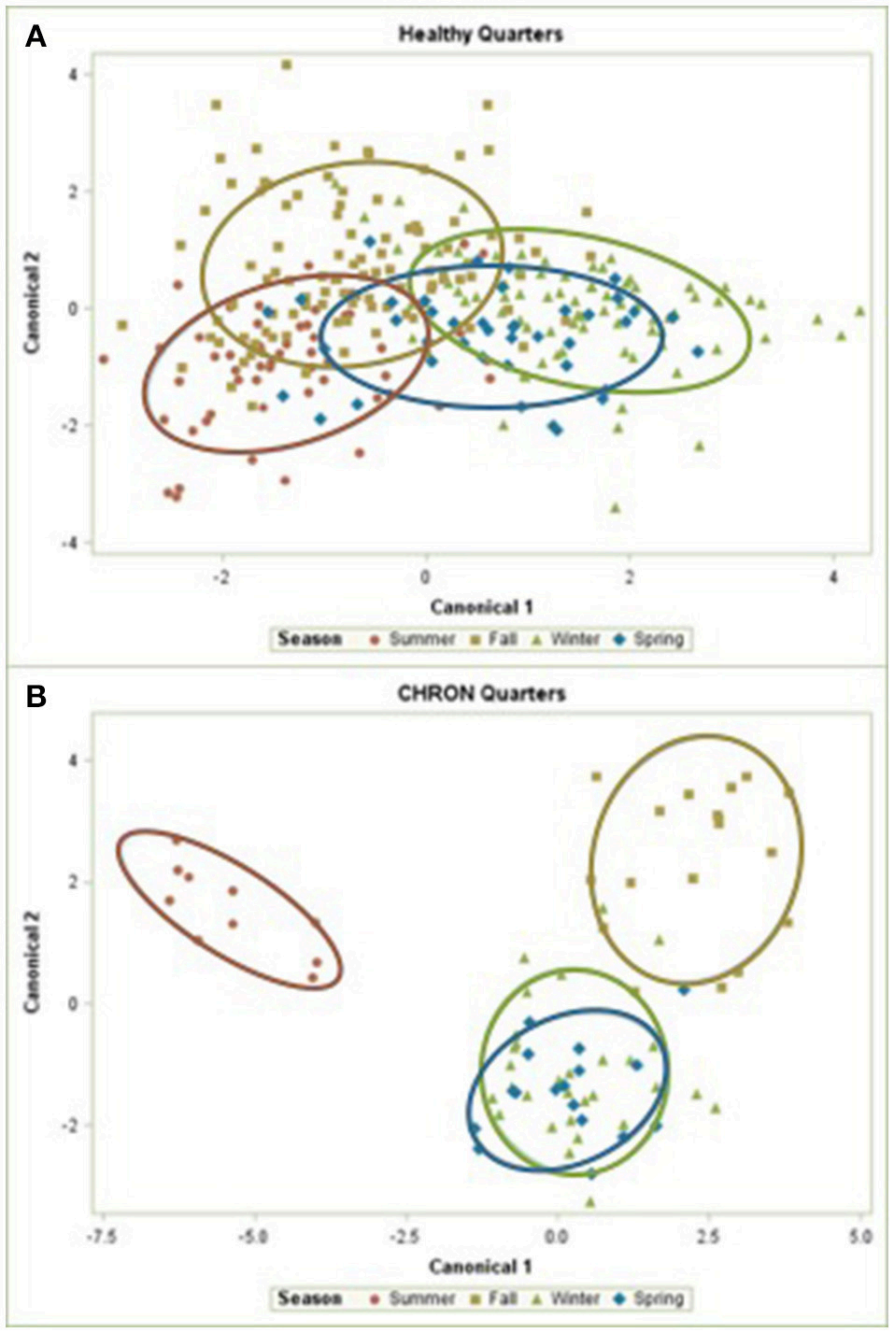
Canonical discriminant analysis of the relative abundances of the top 50 OTUs in Healthy or CHRON bovine milk samples by season. Each symbol represents one milk sample. Healthy, Quarters that had culture-negative milk with SCC < 100,000 cells/mL at DR, C1, and C2; CHRON, Chronically inflamed quarters that had culture-negative milk samples with SCC ≥ 150,000 cells/mL at DR and C1; Sampling periods, DR, dryoff, C1, first week of lactation, C2, second week of lactation.

**Figure 6:**
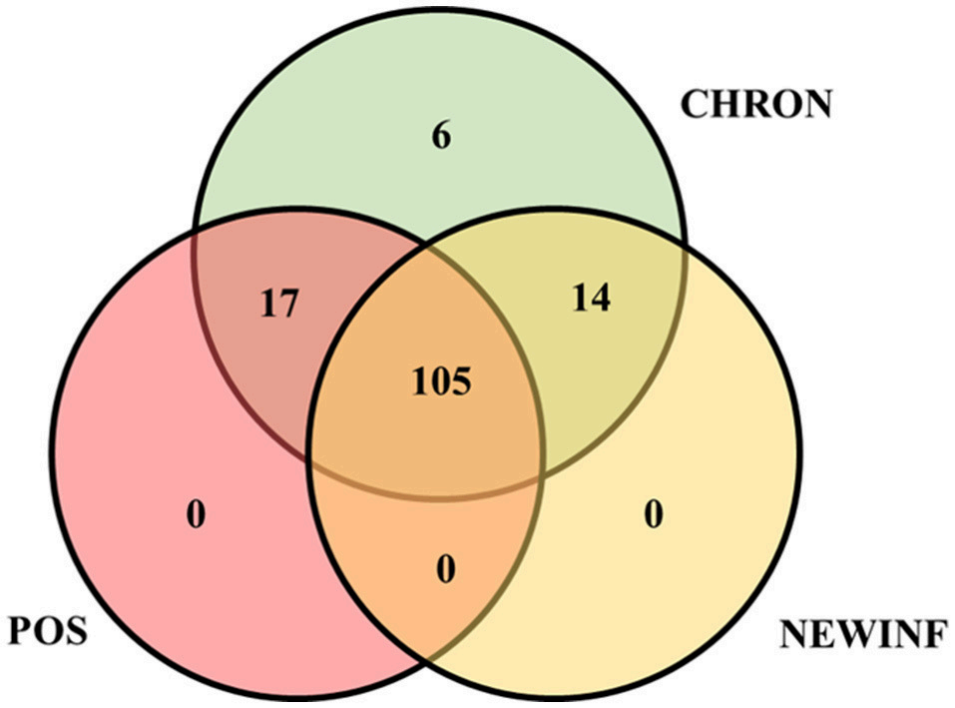
Venn diagram of the 142 most prevalent OTUs found in milk samples collected from inflamed mammary quarters from dryoff to 150 DIM. CHRON, Quarters (*n* = 17) that had culture-negative milk samples with SCC ≥ 150,000 cells/mL at DR and C1; NEWINF, Quarters (*n* = 6) that had new culture-negative inflammation with variable DR milk sample results and culture-negative milk samples with SCC ≥ 150,000 cells/mL at C1 and C2; POS, Quarters (*n* = 3) that had bacterial growth in culture and SCC ≥ 150,000 cells/mL at DR, C1, and C2; Sampling periods, DR, dryoff, C1, first week of lactation, C2, second week of lactation.

The 142 most common OTUs comprised 99.9% of the total sequences across all samples (Figure 3). Among the top OTUs, g_*Staphylococcus* (*P* = < 0.0001), g_*Knoellia* (*P* = 0.041), f_*Aerococcaceae* (*P* = 0.0083), and g_*Coxiella* (*P* < 0.0001) were associated with cohort (Table 3; Figure 4). The Positive cohort had the greatest prevalence of *Staphylococcus* sequences, with 16% of the sequences belonging to this genus. In contrast, only 0.75% of the sequences in the Healthy samples were classified to the genus *Staphylococcus* (Figure 4). The prevalence of *Coxiella* sequences was greater in New Inflammation quarters (6.8%) than in any other cohort (0.13–1.2%) (Table 3; Figure 4). In sum, 11 of the top 20 OTUs comprising 1% or more of the total sequences varied seasonally (Table 3). These OTUs included unclassified Bacteroidetes (*P* = 0.0002) and *Enhydrobacter* (*P* = 0.0035) (Figure 4). Overall community composition varied seasonally within Healthy quarters and also within CHRON quarters (Figure 5). The prevalence of some OTUs varied by stage of lactation, but this variation was less significant than the variation associated with season (Table 3). Among the 3 inflamed cohorts (CHRON, NEWINF, and POS), 105 of the top 142 OTUs were found in all cohorts and only the CHRON cohort had unique OTUs (Figure 6).

**Table 3:** *P*-values for top OTU associations with Cohort, Season, or Sample Period.

**OTU**	***P-*****value**		
	**Cohort**	**Season**	**Sample period**
p_Bacteroidetes	*	****	***
g_Enhydrobacter	*	**	*
g_Acinetobacter	*	*	*
g_Staphylococcus	****	*	*
g_Janthinobacterium	*	*	*
g_Corynebacterium	*	****	**
f_Ellin6075	*	****	**
g_Fibrobacter	*	****	*
g_Knoellia	**	**	**
g_Cupriavidus	*	**	***
g_Pantoea	*	*	*
g_Arthrobacter	*	****	*
f_Aerococcaceae	***	***	*
g_Aerococcus	*	*	**
g_Coxiella	****	*	*
f_Rhodocyclaceae	*	****	****
o_Solibacterales	*	***	*
g_Brevundimonas	*	**	*
g_Psychrobacter	*	*	*
g_Burkholderia	*	*	*

* P > 0.05.

** 0.05 ≥ P > 0.01.

*** *0.01 ≥ P > 0.001*.

**** P ≤ 0.001.

*Cohort, Healthy, Quarters (n = 80) that had culture-negative milk with SCC < 100,000 cells/mL at DR, C1, and C2; CHRON, Quarters (n = 17) that had culture-negative milk samples with SCC ≥ 150,000 cells/mL at DR and C1; NEWINF, Quarters (n = 6) that had new culture-negative inflammation with variable DR milk sample results and culture-negative milk samples with SCC ≥ 150,000 cells/mL at C1 and C2; POS, Quarters (n = 3) that had bacterial growth in culture and SCC ≥ 150,000 cells/mL at DR, C1, and C2; Sampling periods, DR, dryoff, C1, first week of lactation, C2, second week of lactation, M2, second month of lactation, M3, third month of lactation, M4, fourth month of lactation, M5, fifth month of lactation*.

#### Culture and sequencing

All samples from the Healthy cohort were culture-negative at DR, C1, and C2. All CHRON samples were culture-negative at DR and C1, and all NEWINF samples were culture-negative at C1 and C2. The samples from the POS cohort were culture-positive at all 3 enrollment samples. Of the 56 culture-positive milk samples, 40 were successfully sequenced (71.4%). Of these 40, 3 had growth of yeast in culture and were not subjected to a comparison of sequencing results and culture results. For milk samples with positive bacterial growth in culture, culture and sequencing results were generally in agreement, with one of the top 5 OTUs matching culture results for 24 of 37 (64.9%) culture-positive milk samples. Only 3 (8.1%) milk samples did not have the cultured bacteria represented in the top 20 OTUs detected from sequencing.

## Discussion

To our knowledge, this is the most extensive longitudinal study of the dairy cow milk microbiota to date, as other studies have only monitored animals from dryoff to 7 DIM (23) or for 2 weeks during lactation (14). Here, we sampled cows from a sand-bedded university research herd that is similar in size and production to an average Wisconsin, USA dairy farm (24). Dairy cows spend a significant portion of their time lying down with their udders in contact with bedding (25), which is known to be densely populated with bacteria (26, 27). Sand is a commonly used bedding material that generally has lower bacterial counts in culture than other bedding materials, such as manure solids, due to the lower amount of available organic matter (12, 26). As expected, the Healthy cohort quarters had very low SCC throughout lactation and a low incidence of CM (16), providing us with an opportunity to study the microbiota of healthy milk through the first half of lactation. This observational study was conducted in a low-SCC herd, which resulted in the uneven distribution of quarters into cohorts.

Importantly, we cultured a large volume of milk (100 μL) because we wanted to maximize specificity when determining which samples were culture-negative (28), rather than diagnose intramammary infection. Previous reports did not culture all milk samples used for microbiota analysis (14, 29). Culturing is an important way to verify milk samples are not contaminated, as sampling within a milking parlor or barn is difficult with contamination rates in mastitis studies reaching nearly 20% (4, 6). The culturing methods recommended by the National Mastitis Council are limited to aerobic cultures near bovine physiological temperature for up to 48 h (3, 20). We could not determine whether samples were culture-negative because the bacteria are unculturable in our culture conditions, or whether the samples were culture-negative because the bacteria we detected with sequencing were non-viable. Many of the most prevalent OTUs found in this and other milk microbiota studies will not grow in commonly used culture conditions and may require colder temperatures (30) or additional nutrients not found in milk (31). Future milk microbiota research could incorporate additional culturing methods to determine if some bacteria detected using sequencing are viable when cultured using appropriate conditions. For example, many of the OTUs we detected likely have a slow doubling time at the normal temperature found within the bovine mammary gland (12, 32).

The lack of growth in culture, combined with our low sequencing success rate indicates low concentrations of bacterial DNA in our milk, which supports the theory that the milk microbiota is not a highly prolific bacterial community (15). The high cycle number required to achieve amplification is similar to the 35 (14, 29) and identical to the 40 (12, 33) cycles used in previous studies. The 53% sequencing success rate for our conventionally collected healthy milk samples is similar to the 40% sequencing success rate in our previous study (12). A previous study also reported difficulty in amplifying DNA from milk but did not report the rate of sequencing success (29), which would have been a valuable metric for comparison. We suggest that this metric should be reported in future studies. The greater sequencing success in milk samples collected from quarters with a history of inflammation and high cycle number required for PCR suggests that the healthy milk microbiota is minimal and does not have the high bacterial abundances found in other bovine sites. Bacteria in the rumen are found at approximately 2.7 × 10^9^ cfu/g in rumen liquids and up to 5.6 × 10^11^ cfu/g in rumen solids (8). This density far outnumbers that of individual quarter-milk samples, although one study reported 10^2^ to 10^5^ copies of the 16S rRNA gene in milk but did not specify the concentration of the gene copies (14). With such low concentrations of bacterial DNA, we may be detecting DNA that has been imported to the mammary gland within leukocytes (33), DNA from bacteria that were phagocytosed by leukocytes upon infiltrating the mammary gland via the teat canal, or DNA from bacteria that are trapped within the keratin of teat canal (15).

Seasonal changes in richness, tendencies toward seasonal changes in diversity, and overall community composition changes across seasons implicate bedding as a potential source of exposure for bacterial DNA found in milk. Bacteria counts in cultured bedding samples are often greater in summer than in winter (27, 34). Previous studies have also reported that bacterial richness increases in summer and that greater richness is often associated with improved health status (10, 14, 35), but mastitis incidence often increases in summer, likely due to increased exposure to pathogens (18). Similar seasonal trends observed in our cohorts support an external source of bacterial DNA found in the mammary gland that is universal among cows, regardless of inflammation status, as all cows were housed on the same farm and exposed to similar environmental conditions. Exposure to bacteria in bedding has been shown to be associated with milk microbiota composition, as overall bacterial community composition differs among bedding types in milk samples collected directly from the cistern of the mammary gland (12). As such, bedding should be investigated longitudinally with culture-independent methods to record seasonal changes in bacterial community composition and compare these changes in the milk microbiota.

We also found differences in richness, diversity, and community composition among quarters with different inflammation status, suggesting possible cow-related sources of bacterial DNA in milk. In humans, orally administered probiotic strains of *Lactobacillus salivarius* or *Lactobacillus fermentum* can later be cultured from milk (36). Dendritic cells have been suggested as a vehicle for transporting bacteria from the gut to the mammary gland in humans (37) but this pathway has not been demonstrated in cattle. The major OTUs we found, such as unclassified Bacteroidetes, *Enhydrobacter*, and *Acinetobacter*, have all been reported in the bovine gut (9, 38). These gut-associated OTUs did not differ in relative abundance among cohorts, but did change seasonally (unclassified Bacteroidetes, *Enhydrobacter*) or across lactation (unclassified Bacteroidetes) (Figure 4). Unclassified Bacteroidetes was a major OTU that varied in prevalence across lactation, with an increase from DR to C1 and C2, followed by a dramatic reduction in prevalence after calving. The change in unclassified Bacteroidetes prevalence may be related to the compositional changes between colostrum and mature milk (39) or these changes in prevalence may be related to dry cow therapy or physiological factors during the transition period.

All quarters of all cows in our study were treated with an intramammary antimicrobial and a teat sealant at dryoff. Dry cow therapy is standard on more than 75% of US dairy farms as a method for curing existing intramammary infections and preventing new infections that may occur during the dry period (40, 41). The dry cow antimicrobial used in our herd contains penicillin and dihydrostreptomycin and is labeled for treatment and prevention of infections caused by *Staph. aureus*, but is expected to be effective against many Gram-positive pathogens (42). The most prevalent OTUs we detected are not included in the labeling for this drug, but we cannot determine whether antimicrobial therapy has an effect on the prevalence of these OTUs because every quarter was treated.

The greater richness and diversity we found in milk collected from quarters with lower SCC and better health outcomes across lactation is consistent with previous reports comparing milk collected from glands with CM to milk collected from apparently healthy glands (10, 13, 14), and with a report comparing the microbiota of milk collected from quarters that had previously experienced CM to milk collected from quarters that had not (35). As with better-characterized systems, a small number of OTUs did not dominate the healthier systems (43, 44). Our richness and diversity values were lower than those reported in other studies with different sequencing and analysis methods (13, 14), but the richness and diversity of our Healthy quarters were similar to healthy first-lactation cows that were subjected to the same sequence and analysis pipeline as the milk samples used in this study (12).

The OTUs reported in healthy milk from other studies include *Faecalibacterium, Lachnospiraceae, Propionibacterium*, and *Aeribacillus* (10, 11). *Lachnospiraceae, Propionibacterium*, and *Faecalibacterium* comprised well under 1% of the total sequences in our milk samples, while *Aeribacillus* was not detected at all. We did, however, detect DNA of known contaminant *Pseudomonas* in our negative controls and removed this from our sample results (12, 45). *Pseudomonas* has been reported as a prevalent OTU in healthy milk samples that were subjected to whole-genome amplification prior to 16S rDNA-targeted PCR (13) and may have been a contaminant rather than a genuine contributor to the milk microbiota. The differences in diversity and OTUs that we detected in milk illustrate that a “core” microbiota cannot be assumed for dairy cow milk, especially when other studies use different methods and report different bacterial composition. Studies of the human microbiota have used standardized methodologies (46) and we suggest that the development of such methods for dairy milk microbiota analysis would help resolve the vast differences observed across different studies.

An unexpected finding in our study was the presence of *Coxiella* in the milk microbiota, as this genus contains a single species, *Coxiella burnetii*, which causes the zoonotic disease Q fever (47). *Coxiella* sequences were found in the NEWINF quarters, with 6.7% of NEWINF sequences belonging to *Coxiella*. A 2011 human outbreak of Q fever, which can cause endocarditis or atypical pneumonia (48), was linked to consumption of raw cow milk (49). The majority of infected cattle show no signs of disease, even when shedding bacteria (48). Those animals in our study with a high prevalence of *Coxiella* sequences did not exhibit signs of clinical mastitis or abortion, according to herd records, but we cannot exclude the occurrence of this disease in our herd, as Q fever is difficult to diagnose in dairy cows (48).

In addition to having decreased SCC compared to the other inflamed cohorts (NEWINF and POS), milk samples collected from quarters in the CHRON group were the only inflamed cohort to contain unique OTUs. These unique OTUs included the rumen-associated genera *Oscillospira* (50), *Clostridium* (51), and *Selenomonas* (52), and each were present at < 1% of the total sequence abundance in CHRON milk samples. In this study we were unable to determine if the microbiota caused changes in SCC or vice versa; we could only determine that the two are associated. *Clostridium* spp., an unexpected member of the milk microbiota, can be found in the rumen (51), but are more often associated with bulk tank milk and dairy farm soil rather than aseptically collected quarter-milk samples. *Clostridium* spp. can survive pasteurization and should be kept to a minimum in milk, especially milk intended for processing into infant formula (53). Like many other OTUs we identified, these ruminal species are difficult to grow in culture (51, 52) relative to the common mastitis pathogens for which our culture techniques were developed (20).

With the NMC culturing techniques we used, we were able to culture bacteria such as non-*aureus Staphylococcus* spp. and *Streptococcus-*like organisms. The 16S rRNA gene sequencing we used provided a greater resolution of bacterial identification for some of our cultured organisms, including *Streptococcus*-like spp. which were identified with sequencing as g_*Lactococcus*, g_*Enterococcus*, or g_*Streptococcus*. This genus-level resolution of *Staphylococcus* spp. is less beneficial than the biochemical identification we used. Although biochemical identification will not distinguish among non-*aureus Staphylococcus* spp., but can distinguish between *Staphylococcus aureus* and other *Staphylococcus* spp. The distinction between *S. aureus* and non-*aureus Staphylococcus* spp. is critical on a dairy farm due to the contagious nature of *S. aureus*. Other researchers have generally found concordance between culture results and sequencing results in culture-positive milk samples (11, 14, 54). Though the milk microbiota is sparse, associations exist between the microbiota detected with next-generation sequencing and health outcomes in the mammary gland.

## Conclusions

The bovine milk microbiota is associated with inflammation status from dryoff through the first 150 DIM, but the milk microbiota is sparse in the healthiest bovine mammary quarters. The microbiota is more abundant but less diverse in mammary quarters that have inflammation or a history of inflammation. The cow population and housing of cows should be described in studies of the milk microbiota so that researchers can gain a better understanding of how the milk microbiota is related to cow and environmental factors. The differences in prevalent OTUs of the milk microbiota across studies indicates that methodologies for examining the milk microbiota should be compared to examine whether differences in results are related to laboratory methods or to the cow populations. Many types of bacteria that have not been previously associated with milk or mastitis are now potential targets for future research into the milk microbiota.

## Ethics statement

This study was carried out in accordance with the recommendations of Protocol Number A01548-08-13, University of Wisconsin-Madison Institutional Animal Care and Use Committee. The protocol was approved by the University of Wisconsin-Madison Institutional Animal Care and Use Committee.

## Author contributions

LH, GS, and PR designed the study. LH, GS, and PR provided experimental and laboratory resources. SM conducted the study, sample collection, and DNA extraction. All authors developed PCR and sequencing methods. JS performed library construction and sequencing. SM performed data analysis. SM, LH, GS, and PR interpreted results. SM wrote the manuscript. LH, GS, and PR reviewed and edited the manuscript. All authors read and approved the final manuscript.

### Conflict of interest statement

The authors declare that the research was conducted in the absence of any commercial or financial relationships that could be construed as a potential conflict of interest.

## Supplementary material

Figure S1 Weekly log10SCC for enrolled quarters. Values with different superscripts (a–d) differ.

Figure S2 Survival to clinical mastitis for enrolled dairy cow mammary quarters.

Figure S3 Negative control sequences prior to contamination removal.
