# Supplementary material for: A Cohort Study of the Milk Microbiota of Healthy and Inflamed Bovine Mammary Glands From Dryoff Through 150 Days in Milk
Source: Front Vet Sci. 2018 Oct 9;5:247. doi: 10.3389/fvets.2018.00247 (PMC6189514; doi:10.3389/fvets.2018.00247)
Supplement: Supplementary file 1 [file Data_Sheet_1.docx]

Table S1. Enrollment criteria for bovine mammary gland quarters.

| Cohort | DR SCC | DR Culture Results | C1 SCC | C1 Culture Results | C2 SCC | C2 Culture Results |
| --- | --- | --- | --- | --- | --- | --- |
| Healthy | < 100,000 cells/mL | negative | < 100,000 cells/mL | negative | < 100,000 cells/mL | negative |
| CHRON | ≥ 150,000 cells/mL | negative | ≥ 150,000 cells/mL | negative | variable | variable |
| NEWINF | variable | variable | ≥ 150,000 cells/mL | negative | ≥ 150,000 cells/mL | negative |
| POS | ≥ 150,000 cells/mL | positive | ≥ 150,000 cells/mL | positive | ≥ 150,000 cells/mL | positive |

Milk samples were collected from all quarters of all cows at the final milking of a lactation (DR), in the first week post-calving (C1), and in the second week post-calving (C2) and analyzed for somatic cell count (SCC) and microbiological culture.

Table S2. Final logistic regression model for sequencing success.

|  | | | | | | 95% CI | |
| --- | --- | --- | --- | --- | --- | --- | --- |
| Predictor |  | β | Standard Error | *P*-value | Odds Ratio | Lower | Upper |
| Intercept |  | 0.474 | 0.221 | 0.032 |  |  |  |
| Cohort |  |  |  |  |  |  |  |
| Healthy | Reference |  |  |  |  |  |  |
| CHRON |  | 0.584 | 0.221 | 0.008 | 1.79 | 1.16 | 2.77 |
| NEWINF |  | 1.52 | 0.459 | 0.001 | 4.56 | 1.85 | 11.2 |
| POS |  | 0.577 | 0.476 | 0.225 | 1.78 | 0.701 | 4.52 |
|  |  |  |  |  |  |  |  |
| Time |  |  |  |  |  |  |  |
| DR |  | -0.419 | 0.296 | 0.158 | 0.043 | 0.043 | 0.043 |
| C1 |  | -0.121 | 0.305 | 0.690 | 0.886 | 0.487 | 1.61 |
| C2 |  | -0.328 | 0.298 | 0.271 | 0.720 | 0.402 | 1.29 |
| M2 |  | -0.613 | 0.302 | 0.043 | 0.542 | 0.299 | 0.980 |
| M3 |  | -0.087 | 0.302 | 0.774 | 0.917 | 0.508 | 1.66 |
| M4 |  | -0.851 | 0.300 | 0.005 | 0.427 | 0.237 | 0.769 |
| M5 | Reference |  |  |  |  |  |  |
