# Supplementary figures and images for: A Cohort Study of the Milk Microbiota of Healthy and Inflamed Bovine Mammary Glands From Dryoff Through 150 Days in Milk
Source: Front Vet Sci. 2018 Oct 9;5:247. doi: 10.3389/fvets.2018.00247 (PMC6189514; doi:10.3389/fvets.2018.00247)

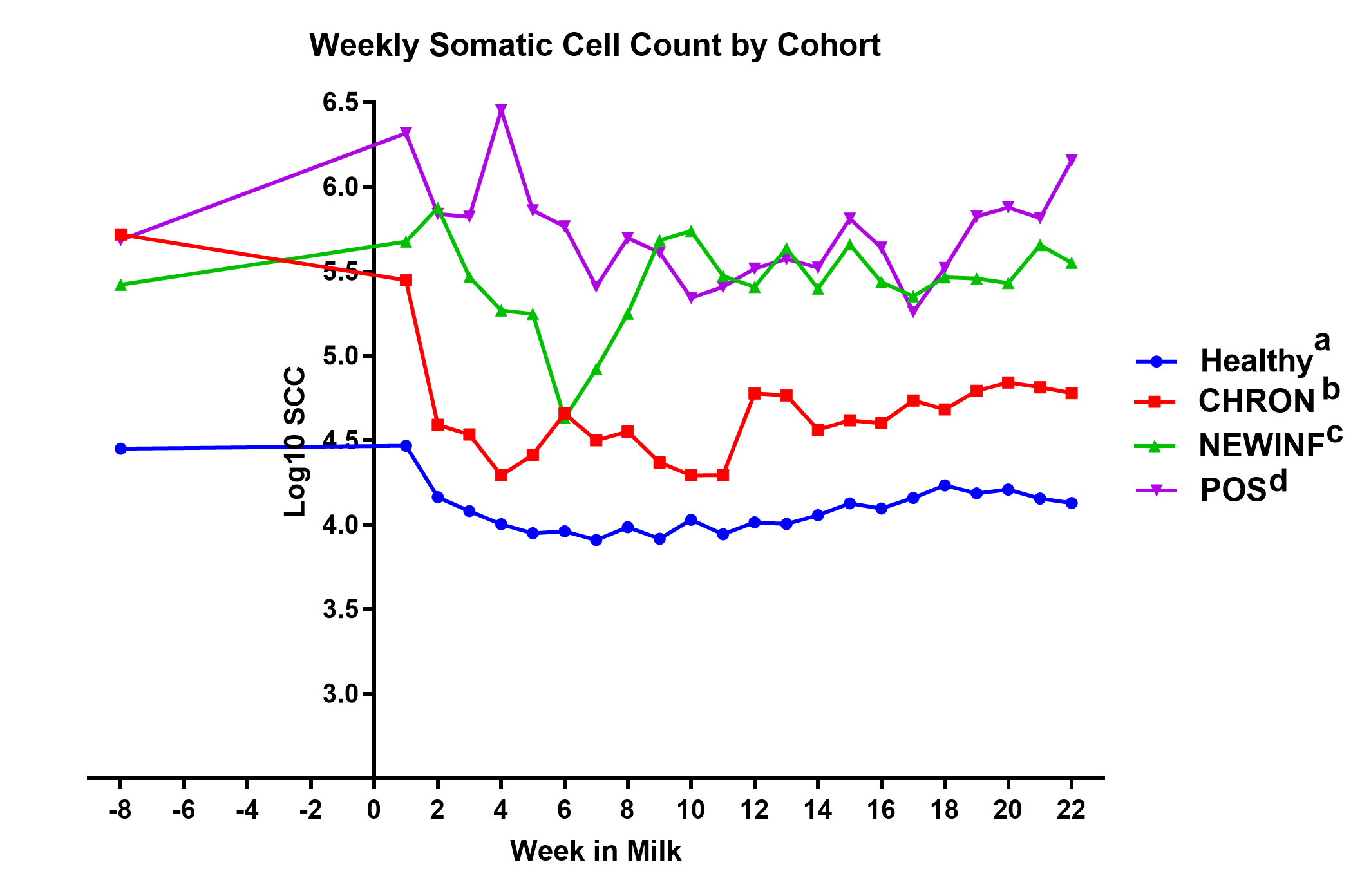

Supplement: Figure S1 — Weekly log10SCC for enrolled quarters. Values with different superscripts (a–d) differ. [file Image_1.TIF]

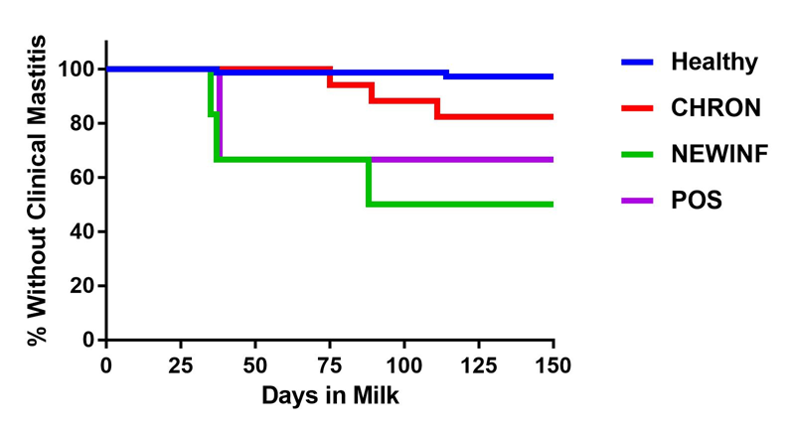

Supplement: Figure S2 — Survival to clinical mastitis for enrolled dairy cow mammary quarters. [file Image_2.TIF]

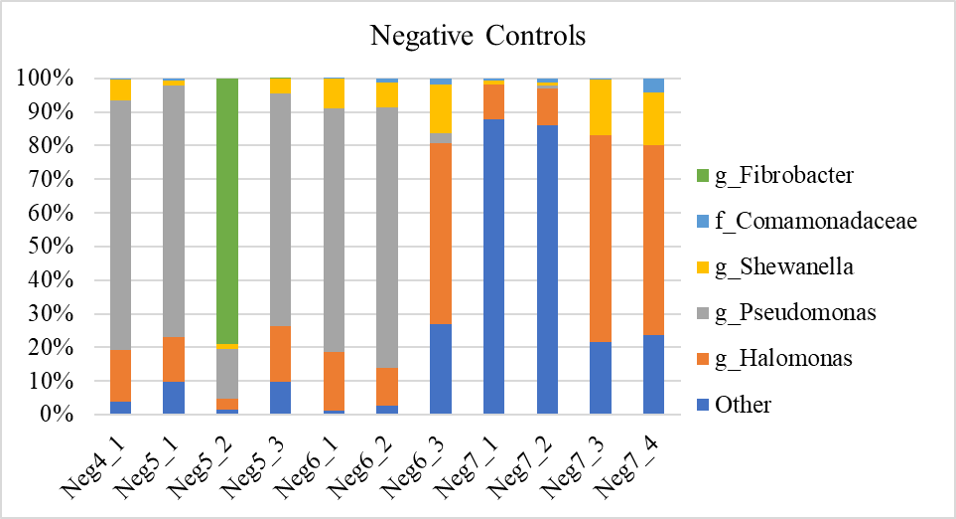

Supplement: Figure S3 — Negative control sequences prior to contamination removal. [file Image_3.TIF]
